# Calibration Technique of a Curved Zoom Compound Eye Imaging System

**DOI:** 10.3390/mi10110776

**Published:** 2019-11-13

**Authors:** Fengli Liu, Xiaolei Diao, Lun Li, Yongping Hao

**Affiliations:** 1 Department of Mechanical Engineering, Shenyang Ligong University, Shenyang 110159, China; DIaohr788@163.com; 2 Technology Center of CAD/CAM, Shenyang Ligong University, Shenyang 110159, China; ll408907652@163.com (L.L.); yphsit@126.com (Y.H.)

**Keywords:** calibration, curved zoom compound eye, Gauss fitting method, optical imaging

## Abstract

A calibration method for the designed curved zoom compound eye is studied in order to achieve detection and positioning of spatial objects. The structure of the curved zoom compound eye is introduced. A calibration test platform is designed and built based on the image characteristics of the compound eye, which can be constructed in the large field view for the calibration target. The spot images are obtained through image processing. The center of the spot is calculated by Gauss fitting method. This method is highly simple and intuitive, and it can be used in a zoom surface compound eye without any complex procedures. Finally, the corresponding relationship between the spot center coordinates and the incident light vector of the corresponding sub-eye is established, and the calibration of the multi vision positioning system is completed.

## 1. Introduction

A biological compound eye has the characteristics of small volume, large field of view, sensitive to moving objects, and so on. [1]. Through the continuous exploration of the structure and imaging mechanism of the biological compound eye, the bionic compound eye imaging system has gradually developed for the intelligent, lightweight and miniaturization, without limitation of the volume to the field angle. With the development of science and technology, the fabrication precision of the artificial compound eye is getting higher and higher, which makes the application field of the bionic compound eye wider [2]. For example, using the large field of view of the compound eye to detect the flying birds near the airport, in order to provide an early warning for the takeoff and landing of the aircraft, using the compound eye, and the phased array radar for compound guidance, and using the compound eye to obtain the relative position and posture of the satellite by mutual measurement in the satellite formation flight, providing data for the synthesizing data [3]. The application of biomimetic technology of insect compound eyes to a series of studies as mentioned above will be a new topic with far-reaching significance and wide application prospects compared with the traditional imaging system [4]. As an important research subject in biology, optics, electronics, information processing, data integration, and automatic control fields, the artificial eye is also widely used in the fields of defense science, medical instruments, and engineering measurement. Therefore, the bionic compound eye has a revolutionary influence on the development of machine vision [5,6]. Zoom surface compound eye is a new type of a non-uniform curved spherical artificial compound eye structure with variable focal length. It allows the artificial compound eye to zoom in on a certain range and effectively solve the problem of poor edge-imaging quality. Compared with the existed binocular positioning method, the compound eye positioning accuracy will be increased because the target can be captured by multiple sub-eyes [7]. At the same time, the positioning scope will be creased from 90° to 120° because the marginal sub-eyes on the bottom of the curved shell can capture the target too.

The main contributions of this paper are as follows: (1) the proposal of a cost-effective curved zoom compound eye calibration that is simpler than that of the previous work [8] based on the method, (2) determination of the calibration mathematical model by establishing the relationship between the spot center coordinates and the incident light vector of the corresponding sub-eye using Gaussian fitting method, (3) applying the Gaussian fitting method on such a small size compound eye with 61 sub-eyes for the first time, and (4) validation of the proposed approach through simulations as well as real experiments.

**Figure 1:**
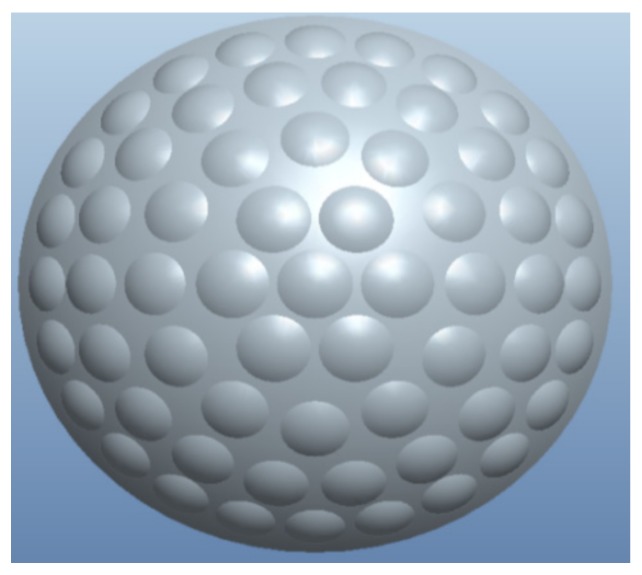
Curved zoom compound eye model.

A curved zoom compound eye is designed and manufactured in order to meet the design requirements of small size and large field of view; the curved zoom compound eye model is shown in Figure 1.

In Figure 1, there are 61 sub-eyes with different curvature radiuses on the spherical surface. The outer and inner radius of the spherical shell are 10 mm and 9 mm, respectively, so the thickness of the compound eye is 1 mm and the sphere arc is 108 degrees. The zoom compound eye model of the curved surface can meet the design requirements of a large field of view.

**Figure 2:**
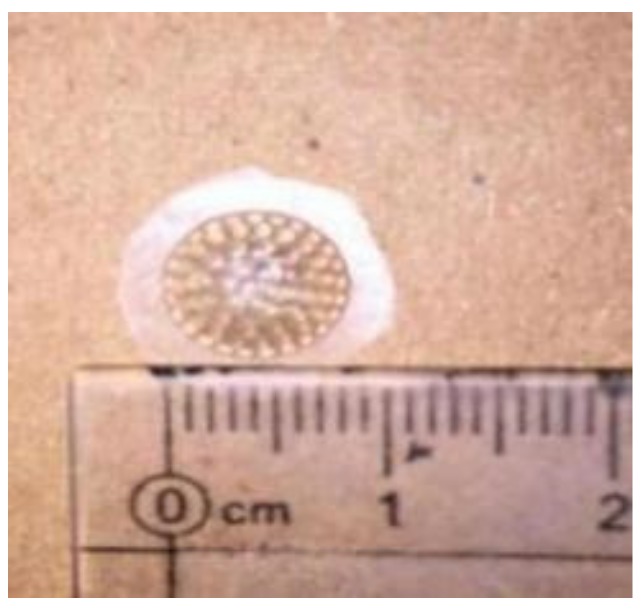
Sample of a compound eye.

The polydimethylsiloxane (PDMS) (Sylgard, 184 silicon elastomer kit) curved compound eye with diameter of 8.66 mm is produced by an injection molding process, as shown in Figure 2. The process cost is low and can be produced in batches compared with the existed manufabrication process [9,10]. The prepared products have good transmittance, flexible materials, good elasticity, and strong chemical stability.

## 2. Calibration of a Curved Compound Eye

### 2.1. Calibration Task

Due to the refraction of sub-eye rays, the target location model of compound eye is divided into two parts. The first part is the part from the target point to the center of each sub-eye, which can be solved by the linear model of the sub-eye center, coordinate, and the angle of the direction vector of the sub-eye. The coordinates of each sub-eye center are fixed in the process of machining the compound eye mold, so the target point can be captured in the market range. The direction vector between the sub-eye can be combined to form a super qualitative equation group, and then the 3D coordinates of the target points are obtained by using the least square method. The second part is the part from the sub-eyes center to the imaging point, which is obtained by calibration [11,12,13]. It needs to find out the angle relationship between the incident ray vector of each sub-eye and the corresponding target image point, so that the angle of the incident ray vector is used as the base for capturing the image point of the target. 

### 2.2. Holistic Calibration of Compound Eyes 

**Figure 3:**
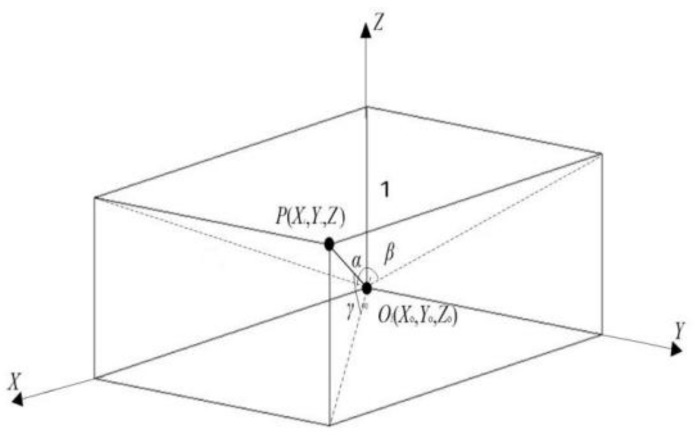
The coordinate relationship between the target and the center of the *i*th sub-eye.

In order to establish the overall calibration of the compound eye imaging system, the relationship between the angle of the incident ray direction of the individual sub-eye, and its corresponding image point must be established firstly, and then the known three-dimensional target can be obtained [14]. The corresponding parameters are set as shown in Figure 3. The target three-dimensional coordinate is set *P*(*X, Y, Z*). The three-dimensional coordinate of the center of the *i*th sub-eye is *O_i_*(*X_0i_, Y_0i_, Z_0i_*). The parameter *α_i_* is the angle between the projection of the connection line from *O_i_* to *P* in the *XOZ* plane and the *x*-axis, while *β_i_* is the angle between the projection of the connection line from *O_i_* to *P* in the *YOZ* plane and the *y*-axis. The three-dimensional coordinate of center of the *i*th sub-eye is *O_i_*(*X_0i_, Y_0i_, Z_0i_*), the connection line between *O_i_* and *P* is *P*(tanα, tanβ, 1), set *a =* tanα, *b* = tan*β*, c = 1, so the connection line between *O_i_* and *P* can be obtained by Equation (1):

(1) $$ai=ci\frac{X-X_{Oi}}{Z-Z_{Oi}}\;bi=ci\frac{Y-Y_{Oi}}{Z-Z_{Oi}}.$$

The direction $P\left(\frac{a_{i}}{c_{i}},\frac{b_{i}}{c_{i}},1\right)$ vector formed by the target point, and each sub-eye is obtained. Thus, the vector incident angle corresponding to each sub-eye can be obtained:

(2) $$\{\begin{array}{c} \alpha i=\arctan\frac{ai}{ci}\\ \beta i=\arctan\frac{bi}{ci} \end{array}.$$

**Figure 4:**
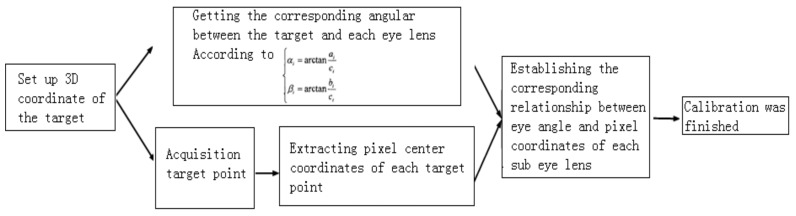
Calibration flow chart of the curved zoom compound eye imaging system.

When there are enough image points to cover the image surface of the entire image detector, the correspondence relationship between the angles of all the incident light rays and the image points in the field of view of the sub-eye can be obtained by Equation (2). The flowchart of the calibration process of a curved zoom compound eye imaging system is shown in Figure 4:

## 3. Calibration Steps of the Curved Zoom Compound Eye

According to experimental conditions, in order to obtain the three-dimensional coordinates of the target, it is necessary to ensure that the horizontal movement axis is perpendicular to the target plane; the target plane is parallel to the plane of the compound eye; the distance from the intersection between the optical axis of the center sub-eye, and the target plane to the center of the whole compound eye is known.

**Figure 5:**
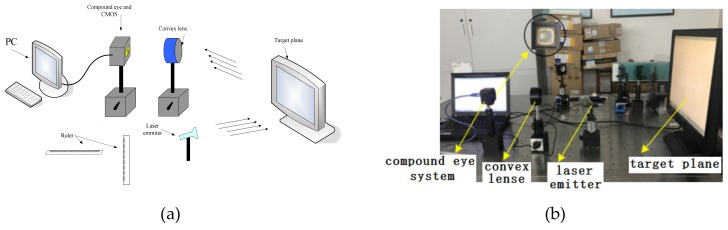
(**a**) calibration test system schematic; (**b**) compound eye positioning test system test bench.

According to the above requirements, a calibration test system schematic is shown in Figure 5a. The experimental equipments required to build the test system as shown in Figure 5b that includes 32-bit notebook computers, curved compound eyes and a complementary metal-oxide-semiconductor (CMOS) detector (CFV301-H2, DO3THINK, Shenzhen, China), laser emitter, and a desktop computer screen. The CMOS optical detection array is used to receive multi-eye imaging of the target point and output the captured image to a notebook computer through USB for further image processing. The focal length of the compound eye center is used as the regulation standard to adjust the distance between the surface of the compound eye and the surface of the CMOS photo detector by assembling the compound eye and CMOS. Thus, all sub-eyes can obtain clear images. Image acquisition was achieved by the CMOS photosensitive surface with a size of 6.55 mm × 4.92 mm, containing 2048 × 1536 effective pixels, each pixel having a unit size of 3.2 μm × 3.2 μm.

By setting the three-dimensional coordinates of the known target light source, the three-dimensional coordinates of the target light source are corrected by using the correspondence relationship between the coordinates of the spot pixel collected in the experiment and the angle of each sub-eye, thereby testing the feasibility of the calibration test system.

### 3.1. Determine the Target Initial Point

**Figure 6:**
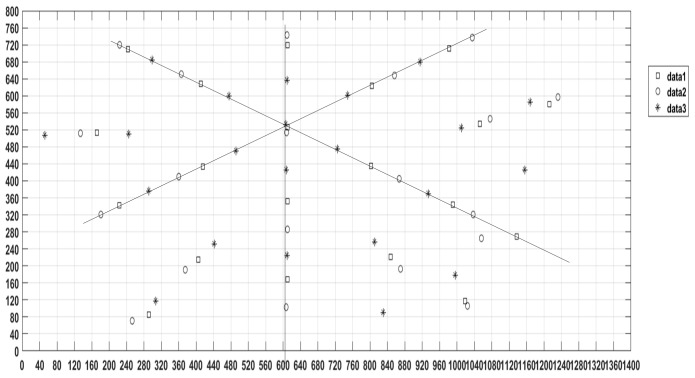
Determines the starting point by the vanishing point.

The initial point of the target is defined as the intersection of the optical axis and the target plane. This point is used as the center to solve the three-dimensional coordinates of the target point. According to the vanishing point principle [15], the compound eye positioning test system is established. By continuously changing the distance between the target plane and the light source, the collected four light spot image planes were overlapped, and the initial point of the target plane was determined as shown in Figure 6. The initial point pixel coordinate of the target plane is (610, 527). There were 33 spots were collected, and the three-dimensional coordinates of the target point can be calculated by the above target positioning model as shown in Equation (1).

### 3.2. Determine the Initial Distance

**Figure 7:**
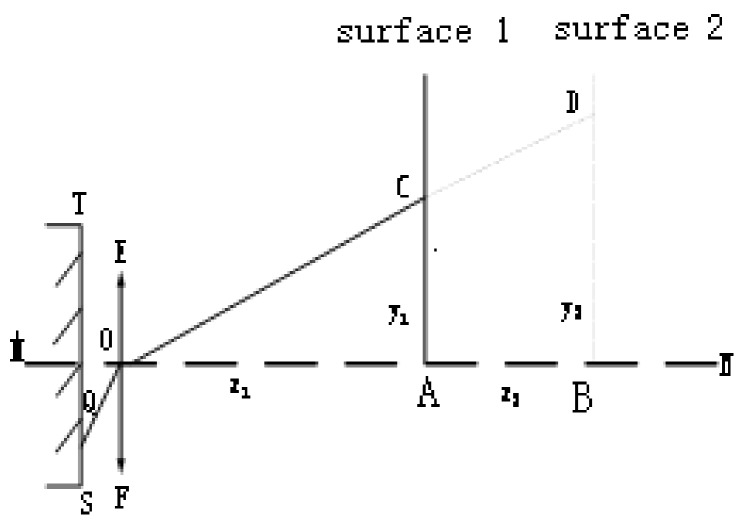
Determines the initial distance schematic.

To obtain the corresponding three-dimensional coordinates of the initial point, that is, the distance between the target plane and the central sub-eye needs to be calculated after determining the two-dimensional coordinates of the initial point of the target [16]. The distance can be obtained by using the triangular geometry formed by the laser beam, the target plane, and the central sub-eye axis with an angle to the target plane. By moving the initial plane, the geometric structures that are similar to each other are formed. The principle structure for determining the initial distance is shown in Figure 7.

In Figure 7: ST is the charge-coupled device (CCD) imaging plane; EF is a central sub-eye; AC (surface 1) is the initial plane; and BD (surface 2) is the plane after moving. According to the similar triangle determination theorem [17]:

(3) $$\frac{z_{1}}{y_{1}}=\frac{z_{2}}{y_{2}-y_{1}}.$$

The parameter *z*_1_ and *y*_1_ are all obtained through the test system after the experiment was conducted. We can get *z*_1_ = 240 mm by Equation (3). The world coordinates corresponding to the target initial point (610, 527) are (0, 0, 240).

## 4. Target Imaging of Multiple Sub-Eye

When there is a target point within the field of view, it can be captured by multiple sub-eyes and a corresponding light spot appearing on the image detector image plane. Experiments are performed with a curved zoom compound eye-targeting test system. Speckle images are acquired and a series of image processing such as graying, binarization, and image filtering are performed on the spot image, so that the coordinates of the spot center can be calculated more quickly and accurately [18,19]. It is possible to establish a reliable correspondence angle relationship between the individual sub-eye and the coordinates of the spot center, thereby the correction of the three-dimensional coordinates of the target point is completed.

### 4.1. Image Binarization

The image is binarized, that is, an appropriate threshold is selected from the gray levels from 0 to 255 gray levels with different brightness levels to obtain a binarized image that is capable of reflecting the overall or local characteristics of the image [20]. Image binarization plays an extremely important role in the fields of image data compression, prominent features, and target recognition, etc. There are only two gray levels left in the images by processing one image with multiple gray levels. The binarization effect of the image directly affects the recognition accuracy of the three-dimensional coordinate point of the target point. Therefore, it is very important to perform the binarization processing of the image on the research subject.

### 4.2. Image Filtering

In the process of collecting images through the photo detector, the combined effects of illumination intensity, atmospheric fluctuations, and inherent noise signals to the photodetector generally result in serious consequences such as distortion of the collected image. Therefore, image noise reduction processing is required in order to extract important target information as much as possible. The existing image noise reduction methods include mean filter, median filter, morphology, BEMD decomposition, higher order statistics, wavelet transform, and Winner filter [16].

### 4.3. Gaussian Fitting Spot Centering Algorithm

**Figure 8:**
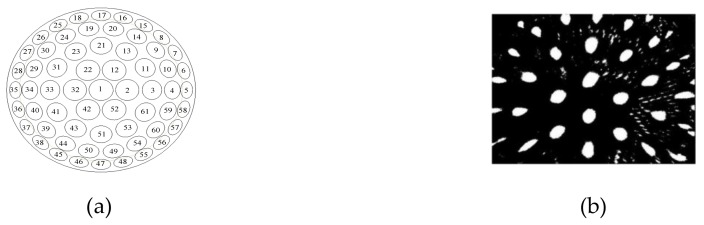
Spot image obtained by image processing; (**a**) sub-eye number schematic; (**b**) spot image when the laser emitter at a specific position.

The laser emitter was removed in the *x*- and *y*-directions. Thus, different spots of the target can be obtained for each sub-eye. One of the images of the spots resulting from image binarization, image filtering, etc. is shown in Figure 8. According to the spot image, the center coordinates of the spot can be calculated [14].

In any section (*x*, *y*) perpendicular to the laser beam, the light intensity distribution is Gaussian and the Gaussian distribution function is:

(4) $$I(x,y)=H\cdot \exp\left\{-\left[\frac{\left(x-x_{0}\right)}{\sigma_{1}^{2}}+\frac{\left(y-y_{0}\right)}{\sigma_{2}^{2}}\right]\right\},$$

where: *I*(*x, y*) is the intensity of the laser beam at its cross-section (*x*, *y*); *H* is the intensity of the spot intensity of its section; (*x*_0_, y_0_) is the spot center coordinates; *σ*_1_, *σ*_2_ are the standard deviations in both directions.

From Equation (4), we can see that the spot center coordinates are the position of the light intensity, so this paper adopts the position of the light intensity as the center coordinate of the spot. Equation (4) is simplified to be a polynomial as shown in Equation (5) by taking the logarithm to the left and right sides of Equation (4):

(5) $$Z=ax^{2}+by^{2}+cx+dy+f.$$

The correspondence of the parameters in Equation (5) is:

(6) $$\{\begin{array}{l} a=-1/\sigma_{1}^{2}\\ b=-1/\sigma_{2}^{2}\\ c=2x_{0}/\sigma_{1}^{2}\\ d=2y_{0}/\sigma_{2}^{2}\\ f=\ln H-x_{0}^{2}/\sigma_{1}^{2}-y_{0}^{2}/\sigma_{2}^{2}\\ z=\ln I\left(x,y\right) \end{array}.$$

Take residual error:

(7) $$\varepsilon_{i}=\left(ax_{i}^{\prime 2}+by_{i}^{\prime 2}+cx_{i}^{\prime }+dy_{i}^{\prime }+f\right)-z_{i}^{\prime },$$

where: (*x*′*_i_*, *y*′*_i_*) is all the coordinates of points on the image surface suitable for Gaussian fitting; *z*′_i_ is the natural logarithm of the gray value of the center of the spot. According to the principle of least squares, the sum of squared residual errors is minimized from Equation (8) to find *a*, *b*, *c*, and *d*:

(8) $$\left[\begin{array}{ccccc} x_{1}^{\prime 2} & y_{1}^{\prime 2} & x_{1}^{\prime } & y_{1}^{\prime } & 1\\ x_{2}^{\prime 2} & y_{2}^{\prime 2} & x_{2}^{\prime } & y_{2}^{\prime } & 1\\ x_{3}^{\prime 2} & y_{3}^{\prime 2} & x_{3}^{\prime } & y_{3}^{\prime } & 1\\ \vdots & \vdots & \vdots & \vdots & \vdots \\ x_{n}^{\prime 2} & y_{n}^{\prime 2} & x_{n}^{\prime } & y_{n}^{\prime } & 1 \end{array}\right]\left[\begin{array}{c} a\\ b\\ c\\ d\\ f \end{array}\right]=\left[\begin{array}{c} z_{1}^{\prime }\\ z_{2}^{\prime }\\ z_{3}^{\prime }\\ \vdots \\ z_{n}^{\prime } \end{array}\right].$$

When the photo detector (CMOS) is used to collect the laser beam spot, when the light intensity is large, or the CMOS exposure time is too long, the gray value easily exceeds 255 and does not reflect the true light intensity. These saturation points are removed because they are easy to generate larger errors if these points are also used Gaussian simulation. The parameters *a*, *b*, *c*, *d*, and *f* are obtained by substituting all points that satisfy this condition into Equation (8). The spot center position (*x*_0_, *y*_0_) and the intensity level *H* are obtained by Equation (9):

(9) $$\{\begin{array}{l} x_{0}=-c/2a\\ y_{0}=-d/2b\\ H=\exp\left(f-c^{2}/4a-d^{2}/4b\right) \end{array}.$$

The light source used in the curved zoom compound eye target calibration system is a laser beam. In an ideal state, the light spot intensity distribution should satisfy the Gaussian distribution. Therefore, the Gaussian fitting method can be used to calculate the center of the spot.

## 5. Establishment of Nonlinear Relationships of Spot Center Coordinates

The coordinate of each light source point in the target plane with the coordinates of the three world coordinates (the center of the sub-eye center of the compound eye) can be calculated according to Equation (10) after the test system is properly adjusted through the test of the compound system of the target point of the curved zoom compound eye:

(10) $$\left[\begin{array}{c} x\\ y\\ z \end{array}\right]=\left[\begin{array}{c} \left(x_{t}-x_{t0}\right)\times 0.212\\ -\left(y_{t}-y_{t0}\right)\times 0.212\\ z_{0} \end{array}\right],$$

where: *x_t_*, *y_t_* are the coordinates of any point in the pixel coordinate system in the target plane; *x*_*t*0_, *y*_*t*0,_ *z*_0_ is the initial distance of the target; *x*, *y* and *z* are the three-dimensional coordinates of the target point in the world coordinate system.

When the three-dimensional coordinates of a target point are known within the field of view, the angle between the target point and the optical axis of the sub-eye that captured the target point can be obtained according to Equation (1) (*α_i_*, *β_i_*, *i* ∈[1,1]), the spot center coordinates (*x_ui_, y_vi_*) are extracted from the collected spot images to obtain a set of correspondences relationship (*α_i_*, *β_i_*) and (*x_ui_, y_vi_*). When enough groups of correspondences relationships were established in the compound eye, multiple sub-eyes in the compound eye are imaged in the imaging surface of the image detector. The unknown target point is to be identified by inverse solving the center coordinates of the spot in the image (*x_ui_, y_vi_*) to its vector angle (*α_i_*, *β_i_*). The linear equation about the incident light in combination with the world coordinates of the sub-eye center of each level is established. After the coordinates (*x_t_*, *y_t_*) corresponding to each sub-eye are obtained by the above method, the three-dimensional coordinates of the world coordinate system can be obtained from Equation (10).

**Figure 9:**
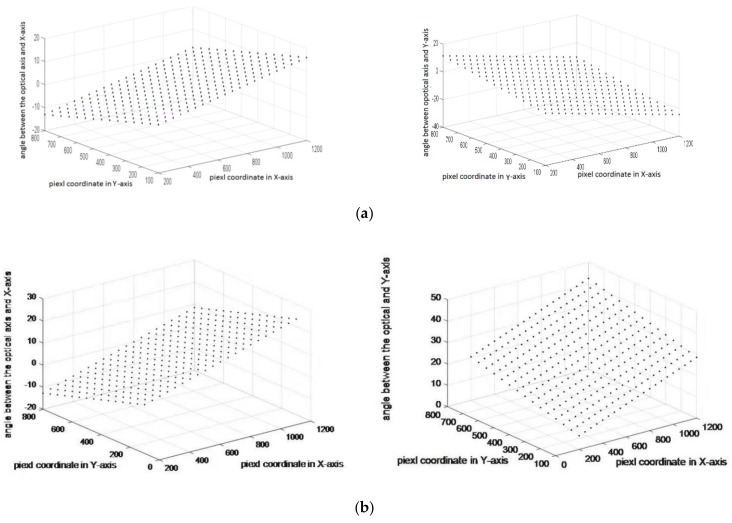
Calibration effect diagram (**a**) No. 32 sub-eye; (**b**) No. 11 sub-eye.

The calibration results can be obtained for each sub-eye by the above method although the captured pixel point numbers are different for each sub-eye. The sub-eye on the top of the compound eye can get more pixel points than the marginal sub-eyes on the bottom. The calibration curved surface positioning test system is adjusted by the corresponding angle relation between the line from the center coordinates of the spot pixel to the target and the optical axis direction vector of the sub-eye [13]. The No. 32 sub-eye and No. 11 calibration effect are shown in Figure 9 as an example. The angles between the connection line from the center of the spot to target and the optical axis projected in the *x*-axis and the *y*-axis are shown in Figure 9a,b, respectively.

The nonlinear relationship between the the optical axis direction vector of the sub-eye lens and the spot center coordinate is finally established to analyze the calibration result of the whole compound eye.

## 6. Conclusions

The calibration task of the curved zoom imaging system is defined, and the whole calibration of the curved zoom imaging system is carried out to determine the initial point and the initial distance of the target. The facula pattern is obtained through the experiment of the curved zoom measuring system, and the image is processed by binarization and filtering. The facula pattern is processed by the Gaussian fitting method to calculate the spot center position. Finally, the nonlinear relationship between the angle and the coordinate of the spot center is established to analyze the calibration results and complete the correction of the compound eye target positioning system.
